# Hospital readmissions of frail older individuals: the challenge of anticipating end-of-life care

**DOI:** 10.3389/fmed.2025.1624555

**Published:** 2025-07-16

**Authors:** Fabien Visade, Chloé Prod'homme, Jean-Baptiste Beuscart

**Affiliations:** ^1^Department of Geriatrics, Universite Catholique de Lille, Lille, France; ^2^Univ. Lille, CHU Lille, ULR 2694 - METRICS: Évaluation des Technologies de Santé et des Pratiques Médicales, Lille, France; ^3^Palliative Care Unit, ULR 2694 METRICS, Centre Hospitalier Universitaire de Lille, Lille, France

**Keywords:** end-of-life, hospital readmissions, advance care plans, older individuals, frail individuals

## Introduction

In an aging population, an increase in the number of patients who are frail and at risk of hospital readmission and death is inevitable. We must find solutions to improve these patients' final moments. Anticipating care is key, but in many countries the traditional approach of advance directives has failed. A continuous multidisciplinary approach based on advance care planning has its merits but comes with many challenges.

## Older patients with repeat hospitalizations

The older adults population represents a variety of clinical profiles, including healthy individuals and others with one or more comorbidities. The latter often experience a loss of functional independence and geriatric syndromes including falls, undernutrition and neurocognitive disorders (1) requiring significant care that generates health expenditure. Recent research has highlighted how clinically diverse the older adults population is. The frailest individuals, which make up a significant proportion of this population, require the most care, including multiple hospitalizations. The risk of hospital readmission is described as a dynamic process increasing with each hospitalization and most often resulting in the patient's death (2). Hospitals are often criticized for being ill-adapted to older adults care (3). Yet in some cases patients hospitalized repeatedly are admitted to units specializing in older adults care (2). Not only is it important to screen patients at risk of hospital readmission and death, but also to ask whether all of these hospitalizations are really necessary. Are they really caused by unstable medical issues? Is there an unidentified underlying social problem, an unorganized care pathway, or an unanticipated medium-term end-of-life situation? And lastly, are all these hospitalizations what the patient and their loved ones want? What these patients need today is a paradigm shift. Other research indicates that end-of-life patients would prefer home care to be with their family (4). Anticipation is also a priority for them, to refocus their care on what they really want (5). Although the patients' profile can make anticipation challenging, advance care planning makes this possible.

## Advance care plans

An advance care plan (ACP) is a process that helps adults of any age or state of health understand, share and discuss their personal values, life goals and preferences for future medical care with their loved ones, carers or caregivers, document them through advance directives and adapt them. The ACP does not replace advance directives, but incorporates them into a more global and adaptable process (6). Strong evidence from systematic reviews shows that an ACP increases the number of advance directives and discussions about future clinical care to help meet patient goals in many populations (6). An ACP can improve patient-clinician communication, reduce unwanted hospital admissions, increase the use of palliative care and improve patient satisfaction and quality of life (7). Above all, this approach serves to provide a personalized care plan to help fulfill the patient's wishes. It facilitates decision-making in emergency situations and when the patient can no longer express their wishes. The final parts of the ACP usually concern hospitalization, resuscitation, artificial nutrition and place of death.

This process can also of course be used for the older adults population. The literature indicates that it is easier to implement an ACP when the older patient has a well-identified terminal-stage disease (8). It is more difficult when it comes to frail patients with multiple comorbidities who are hospitalized repeatedly. Challenges to implementing an ACP in the older adults population include the perceived inability to execute an ACP, finding the right time, the opinion that the patient should initiate the ACP, and not wanting to deprive patients of hope. However, certain methods have proven helpful, such as involving loved ones, training professionals initiating and executing the ACP, providing explanatory materials to patients, communication between hospital and home care professionals, and interdisciplinary collaboration. However, more detailed information on mechanisms to improve ACPs for these patients is needed and will be evaluated in ongoing trials (9).

ACPs have been tested for the older adults in many countries where legislation and the health authorities allow discussions on anticipating care. A crucial factor to consider is the time needed to execute the ACP. This is especially true for frail and sick older patients, as the process can be time-consuming and a single discussion will probably not suffice. Although ACPs are cost-effective in that they reduce the amount of care (such as visits to emergency departments or hospitalizations), the cost is not always covered. However, funding the ACP procedure can improve the use of palliative care for patients who are seriously ill, thus reducing the quantity of care, particularly in hospitals (10). Consideration is needed to adapt ACP billing to the time allocated.

## Including patients with diminished decision-making capacity

Patient involvement is vital when creating an ACP. One of the main challenges with the older adult is involving the patient when their decision-making capacity is diminished. This can lead to major treatment that the person does not want at the end of their life. Most ACP testing has excluded people with neurocognitive disorders. International consensus has led to a specific framework to establish an ACP in cases of neurocognitive disorders, and supports the inclusion of dementia patients and their families, preferably with appropriate documentation and discussions continuing until the disease becomes severe (11). Tools can be used to help engage discussions with dementia patients and their loved ones and improve understanding. However, an ACP for a dementia patient is more likely to be successful if anticipated and initiated before the disease progresses.

## Conclusion

There are many challenges to implementing an ACP for older patients with repeat hospitalizations and at high risk of death. A holistic approach is needed to encompass the ethical, legal, social and economic aspects. Rather than simply discussing end-of-life wishes, we must take into account the specificities of this population. Interdisciplinary collaboration between geriatricians and palliative care will no doubt help to provide better care for these patients.
